# Supplementary material for: Neural and social correlates of attitudinal brokerage: using the complete social networks of two entire villages
Source: Proc Biol Sci. 2021 Feb 10;288(1944):20202866. doi: 10.1098/rspb.2020.2866 (PMC7893238; doi:10.1098/rspb.2020.2866)
Supplement: Supplementary Information [file rspb20202866supp1.docx]

**Supplementary Information for**

Neural and social correlates of attitudinal brokerage: using the complete social networks of two entire villages

**This PDF file includes:**

Supplementary information text

Figures S1 to S11

Tables S1 to S7

SI References

Supplementary information text

**1. Screening tests** Resting-state fMRI (functional magnetic resonance imaging) data (n=194; Village K: n=72, Village L: n=122) were acquired from a subpopulation from the Korean Social Life, Health and Aging Project (KSHAP). To select participants for resting-state fMRI, we first selected 316 individuals via quota sampling based on age group, gender, subjective health, and social network size. Then a line of screening tests was conducted. Following screen criterion ensured to exclude older adults who were cognitively impaired. First, people were examined to confirm if they had significant cognitive or behavioral changes in the past year. They include participants who either scored below 1.5 SD in Mini-Mental State Examination for Dementia Screening (MMSE-DS), below 5 percentiles on Long-term Memory Recall Index or below 5 percentiles in Working Memory Index in Elderly Memory Scale based on age and education specified norm. Based on self-respondent or informant-respondent semi-structured interview of Clinical Dementia Rating (CDR) or Korean Dementia Rating Questionnaire (KDSQ), cognitively impaired (CDR score of 0.5 or 1) were excluded. In turn, 194 participants underwent a functional MRI scan at the Seoul National University Brain Imaging Center. Thirty-five participants were excluded from this study due to excessive scan movement (maximum FD frame-wise displacement > 4.5 mm or mean frame-wise displacement > 0.5 mm), neurological abnormality, or diffuse signal confounds. Also, six were excluded from this study due to unavailability of attitudinal diversity scores of social network members because they did not have any social network member. Finally, resting-state fMRI data of 139 participants were used.

**2. Acquisition of resting-state fMRI data and image processing Following the screening tests,** resting-state fMRI data were acquired from a sub-population of the KSHAP (n = 139; Township K = 47, Township L = 92). Resting-state fMRI data were acquired on a 3T Siemens Trio scanner. During the scan, participants were instructed to rest quietly with their eyes open and not fall asleep. We acquired 300 contiguous EPI functional images (TR = 2000 ms, TE = 30 ms, FOV = 240 × 240 mm, FA = 79°, voxel size 3 × 3 × 3 mm, gap = 1 mm, acquisition time = 10 minutes). In order to acquire high spatial resolution, cerebellar regions were excluded in the acquisition. T1-weighted magnetic prepared rapid gradient echo (MPRAGE) images were acquired (Sagittal slices, slice thickness 1 mm, TR = 2300 ms, TE = 2.36 ms, FOV = 256 × 256 mm, FA = 9°, voxel size 1 × 1 × 1 mm³). The study was approved by and performed in accordance with the relevant guidelines and regulations by the Institutional Review Board of Yonsei University (IRB number: YUIRB-2011-012-01; Township K survey, Township K brain fMRI, and Township L survey) and the Institutional Review Board of Seoul National University (IRB number: 1801/001-003; Township L brain fMRI), and all participants provided written informed consent for the research procedure.

Image preprocessing and denoising was performed using the SPM12 software (Wellcome Department of Imaging Neuroscience, Institute of Neurology, London, UK) with the Conn toolbox 18.a (http://www.nitrc.org/projects/conn) default preprocessing pipeline. Functional images were corrected for motion and slice time and warped into MNI standard space. Images were smoothed with an 8-mm full-width half-maximum Gaussian kernel. In addition, the Artifact Detection Tools (<https://www.nitrc.org/projects/artifact_detect/>) were used to identify motion and signal intensity outlier images. Images with global mean intensity Z-values > 5 and movement > 0.9 mm were identified as outlier images. In a denoising process before adjacency matrix construction, six estimated motion parameters, six first-order temporal derivatives and outlier images were used as nuisance covariates in the time-series linear regression. T1-weighted images were segmented into gray matter, white matter, and cerebrospinal fluid (CSF) and warped into MNI standard space. Based on the aCompCor (anatomical component-based noise correction) method, five principal components each from the white-matter and cerebrospinal fluid (CSF) time series were regressed out to exclude non-gray matter BOLD signal (1, 2). Band-pass temporal filtering (0.008–0.09) was applied to exclude physiological noise.

According to the recent benchmarks of denoising processes, the aCompCor method was one of the top-performing methods in the mitigation of motion confounds (3). We did not control for global signal regression (GSR) because some argue that GSR may introduce artifactual biases and remove potentially meaningful neural components (4, 5). For each subject, mean time series were extracted by averaging all voxels composing each region for each time point from the 227 regions of Shen’s whole-brain parcellation atlas (37 cerebellar regions were excluded) (6). Pearson’s correlation coefficients were calculated between each pair of regions and transformed to Fisher’s Z-scores. Therefore, 139 individual whole-brain connectivity matrices containing 25,651 (= (227 × (227–1))/2) edges were constructed.

**3. Mentalizing brain network** We identified 51 mentalizing-related brain regions among the 227 regions from Shen’s whole-brain parcellation atlas. To do so, we used a mentalizing mask, which is the association test map of the term “mentalizing” from the Neurosynth meta-analytic tool (<http://neurosynth.org/analyses/terms/mentalizing/>). In the association test map, mentalizing-related brain regions were identified by integrating previous studies about mentalizing. Specifically, the association test map was created based on the two-way ANOVA testing for the presence of a non-zero association between “mentalizing” term use and voxel activation in the previous studies. The mentalizing mask included 46,943 voxels in the vicinity of 6,824 voxels consistently activated in 151 studies that frequently used the term “mentalizing,” applying a rigorous FDR statistical threshold of q < 0.01 (7). After downloading the mentalizing mask, we identified 51 “mentalizing” regions out of 227 regions in Shen’s whole-brain parcellation atlas that contain more than 20 voxels in the “mentalizing” mask. All 51 regions are shown in Fig. S5. Given these 51 “mentalizing” brain regions, we identified a mentalizing brain network consisting of 10,251 edges between the 51 “mentalizing” regions and all 227 whole-brain regions, in line with previous studies (8)

**4. Structural brokerage and hub node** It should be noted that a structural brokerage position is conceptually different from a hub node. While a node in a structural brokerage position refers to a node having connections with two people who are not otherwise connected, a hub node indicates a node having many connections or a node that occupies a central position in a network. Therefore, one can be in a structural brokerage position without being a hub node. Table S6 presents correlations between Burt’s structural constraint and hub node measures (e.g., betweenness centrality, closeness centrality, and eigenvector centrality). The correlation coefficients were modest (between -0.636 and -0.188), which implicates that a structural brokerage position and a hub node are conceptually different.

**5. Connectome-based predictive modeling (CPM)** To identify the association between brain connectivity and attitudinal diversity scores, this study applied CPM. CPM is a method based on a machine learning approach to predict individual attributes based on resting-state brain functional connectivity, and the MATLAB code used for CPM is freely available online (<https://www.nitrc.org/projects/bioimagesuite/>) (9, 10). To test this association, CPM generally implements leave-one-out cross-validation (LOOCV) (9). For each LOOCV round, (n – 1) participants are used as the training sample to estimate the predictive model, and the remaining one is used as the test sample to evaluate the predictive accuracy of the model. LOOCV rounds were repeated such that each participant was used once as the test sample.

In each round of LOOCV, a linear regression model wherein brain connectivity predicts attitudinal diversity scores is constructed using a training sample of (n – 1) participants. To do so, Pearson’s correlation coefficients are first computed between the connectivity values of every edge in mentalizing brain connectivity and attitudinal diversity scores among participants in the training sample. Applying a statistical significance threshold of p < 0.01 following previous studies (9, 11-14), we selected edges for which connectivity values were positively or negatively correlated with attitudinal diversity scores. This procedure is called “feature selection” (9). Then, a linear regression model is generated to associate the sum of the connectivity values of the selected edges to attitudinal diversity scores for positively correlated edges and negatively correlated edges. After constructing a linear regression model based on the training sample, the model-predicted attitudinal diversity score of the participant in the test sample is estimated by submitting his/her connectivity value sum of the selected edges to the linear regression model.

To estimate predictive accuracy, which represents the significance of the association, Pearson’s correlation coefficient (r) and mean absolute error (MAE) between the observed attitudinal diversity scores and model-predicted attitudinal diversity scores were calculated. In previous studies, Pearson’s correlation coefficient was widely used to estimate the predictive accuracy of CPM (9, 11-14). However, some argued that Pearson’s correlation coefficient could produce biased and misleading estimates of predictive accuracy, suggesting that MAE could be a more reliable measure of predictive accuracy when Pearson’s correlation coefficients and MAE produce different results (e.g., when one is significant and the other is not) (15).

To account for the nonindependence of the leave-one-out rounds, permutation tests were conducted to estimate the statistical significance of predictive accuracy by randomly shuffling attitudinal diversity scores and re-running the entire LOOCV procedure 1000 times. By repeating the LOOCV procedure that predicts shuffled attitudinal diversity scores, 1000 ‘permuted r’ and ‘permuted MAE’ could be obtained and used as a proxy sampling distribution. Finally, p-values are computed using the following formula: $\frac{1+k}{1001}$, where *k* is the number of permutated r whose values were higher than an observed r, or the number of permutated MAE whose values were lower than an observed MAE (13). Because permutation testing is computationally intensive, permutation tests were implemented on an EC2 instance (m4.4xlarge) of Amazon Web Services (16). To control for confounding effects, we conducted control analyses as described in the Supplementary Information text (7. Control analyses).

In addition to the LOOCV, five-fold cross-validation was also employed (17). The five-fold cross-validation procedure divided all participants into five subsets and used four of the subsets as the training sample and the remaining one as a test sample. By repeating five times, the model-predicted attitudinal diversity scores could be collected for all participants. Considering the possibility that data division affected predictive accuracy, we repeated the five-fold cross-validation 50 times and used the average results as the final predictive accuracy of the model. Finally, the statistical significance of predictive accuracy was assessed based on 1000 permutations.

In CPM (connectome-based predictive modeling) protocol, it is assumed that some edges in the brain connectivity graph can contribute to the positive outcome (i.e., positive network). Simultaneously, some edges can contribute to the negative outcome (i.e., negative network) (9). Therefore, the prediction performance is estimated for the respective network. Thus, in our paper, CPM first identifies a set of edges in mentalizing brain connectivity of which strength contributes to attitudinal diversity (high-diversity network). Then CPM identifies a set of edges in mentalizing brain connectivity of which strength contributes to attitudinal homogeneity (low-diversity network). After that, CPM estimates the predictive performance of the high-diversity network and low-diversity network, respectively. The high-diversity network’s high predictive performance shows that CPM successfully identified edges in mentalizing brain connectivity, which contribute to attitudinal diversity (i.e., diversity was positively predicted by mentalizing brain connectivity). On the other hand, the low-diversity network’s low predictive performance shows that CPM “failed” to identify edges in mentalizing brain connectivity contributing to low diversity (i.e., diversity was not negatively predicted by mentalizing brain connectivity). In short, we could find edges in mentalizing brain connectivity that significantly contribute to attitudinal diversity, which is consistent with our hypothesis. However, we could not find edges in mentalizing brain connectivity that significantly contribute to attitudinal homogeneity.

**6. Strong homophily in social networks by gender role attitudes** To verify our assumption that people having similar attitudes are highly clustered in our social network data, we calculated how many traditional people, on average, each traditional person can reach out through path (i.e. sequence of ties) in the social network. Also, we calculated how many egalitarian people, on average, each egalitarian person can reach out in the social network. Then we compared these numbers to the numbers that would have been observed if we kept the network topology and attitude distribution the same and then randomly reassigned participants to have traditional or egalitarian attitudes. The results showed that the observed numbers were much larger than those expected because of the strong homophily regarding the attitudes both in Township K and L (Fig. S9).

**7. Control analyses** Control analyses were implemented by controlling for the following variables during the “feature selection” procedure of leave-one-out cross validation. In particular, when correlation coefficients were computed between edges and attitudinal diversity scores among participants in the training set, partial correlation coefficients controlling for following variables were computed (9). First, age and sex were controlled for. Second, age and sex were controlled for along with one additional variable among the following variables, in turn: education years, social network size (the number of social network members), average communication frequency (days in which the participant and each social network member communicate during a year on average), Burt's structural constraint, betweenness centrality, close centrality, eigenvector centrality, Mini-Mental State Examination for Dementia Screening (MMSE-DS) score (0~30) (18), subjective health (5-point Likert scale; 1=poor, 2=slightly poor, 3=good, 4=very good and excellent) personality traits assessed using the NEO Five-Factors-Inventory on a 4-point Likert scale (agreeableness, extraversion, neuroticism, opennness to experience, conscientiousness) (19), gender role attitudes (1~5), and village (0=Village K, 1=Village L). In addition to the confounders above, the head motion may cause spurious patterns of brain functional connectivity. The head motions could artifactually increase prediction performance if head motion and attitudinal diversity scores are correlated (9). Therefore, some argue that head motion should be not only corrected in the cleaning pipeline but also included in control analyses (9). In turn, we controlled for maximum frame-wise displacement and mean frame-wise displacement. If predictive accuracy remained significant even after controlling for these variables during the feature selection procedure, we considered that the predictive accuracy was robust.

Previous works suggested that brain activity and connectivity in the mentalizing system is associated with social network position such as structural brokerage (20). Therefore, we examined whether mentalizing brain connectivity was directly correlated to Burt’s structural constraint or other social network position variables (betweenness centrality, closeness centrality, eigenvector centrality). However, mentalizing brain connectivity was correlated with none of these variables at p<0.05 level (Table S6). Also, the association between the mentalizing brain connectivity and attitudinal diversity scores (ambivalence score, standard deviation) remained significant even after controlling social network position variables.

**8. Prediction using whole-brain connectivity** To complement our hypothesis-driven ROI connectivity analyses with a data-driven analytic approach, we used “whole-brain” resting-state functional connectivity to predict attitudinal diversity of social network members. Consistent with the results using mentalizing connectivity, whole-brain connectivity was positively associated with both standard deviations of gender role attitudes among social network members (r = 0.2156, p = 0.031; MAE = 0.8168, p = 0.080) and ambivalence scores (r = 0.1940, p = 0.071; MAE = 0.8466, p = 0.045). Also, just like ROI analysis, whole-brain connectivity was not negatively associated with both standard deviations (r = 0.0442, p = 0.390; MAE = 0.8931, p = 0.592) and ambivalence scores (r = 0.1663, p = 0.122; MAE = 0.8943, p = 0.243). Also, 5-fold cross-validation showed that both standard deviations (r = 0.1586, p = 0.051; MAE = 0.8323, p = 0.082) and ambivalence scores (r = 0.1602, p = 0.037; MAE = 0.8630, p = 0.022) were positively associated with whole-brain connectivity. A set of edges of which connectivity values positively predicted attitudinal diversity scores for more than 90 percent of leave-one-out cross validation (LOOCV) rounds were represented in Fig. S10, which showed that 38.64% of ties in predictive whole-brain network were within mentalizing brain network.

To show that the prediction performance of mentalizing connectivity is above and beyond whole brain connectivity, we used the “lesioned connectivity” approach. The lesioned connectivity refers to whole-brain connectivity excluding edges in mentalizing brain connectivity (21). If the prediction performance of the lesioned connectivity is lower than the prediction performance of mentalizing brain connectivity, we can conclude that mentalizing brain connectivity plays a particularly important role in predicting attitudinal diversity scores. As a result, when predicting the ambivalence score, the prediction performance of the lesioned connectivity (r = 0.1102, p = 0.256; MAE = 0.8846, p = 0.201) was substantially lower than the prediction performance of mentalizing brain connectivity (r = 0.2301, p = 0.046; MAE = 0.8305, p = 0.030). However, when predicting the standard deviation, prediction performance of lesioned connectivity (r = 0.1967, p = 0.074; MAE = 0.8188, p = 0.126) was similar to the prediction performance of mentalizing brain connectivity (r = 0.2033, p = 0.061; MAE = 0.8190, p = 0.114). We can conclude that mentalizing brain connectivity plays a particularly important role in predicting outcome at least regarding ambivalence score.

Next, we included “attitudinal diversity scores predicted by lesioned connectivity” and “attitudinal diversity scores predicted by mentalizing connectivity” in the same regression model to predict observed attitudinal diversity scores. As shown in Table S8, when predicting ambivalence score, the coefficient of mentalizing connectivity was statistically significant (p=0.014) but the coefficient of lesioned connectivity was not statistically significant (p=0.593). And, the coefficient of mentalizing connectivity was marginally higher than the coefficient of lesioned connectivity (p=0.097). When predicting standard deviation, however, both coefficients of lesioned connectivity (p=0.275) and mentalizing connectivity (p=0.212) were not statistically significant. And the coefficient of mentalizing connectivity was slightly higher than the coefficient of lesioned connectivity but the difference between the coefficients was not statistically significant (p=0.929). Thus, it seemed that the predictive accuracy of mentalizing connectivity was marginally higher than the one of the lesioned connectivity at least regarding ambivalence score.

**9. Reproducibility check** To confirm reliability of our results, we conducted a reproducibility check. Given that our participants were from two non-adjacent, independent neighborhoods, Village K and Village L, we tried to examine if the predictive performance of mentalizing brain connectivity in one neighborhood could be successfully replicated in the independent sample of the other neighborhood. First, external validation analyses used Village L sample (n=92) to conduct leave-one-out cross-validation (LOOCV) to identify predictive mentalizing brain network. We identified a set of edges of which connectivity values positively predicted attitudinal diversity scores for more than 90 percent of leave-one-out cross validation (LOOCV) rounds. Note that the identical edges and weights of mentalizing brain connectivity defined in one village are used to predict attitudinal diversity scores in another village. After that, we used the mentalizing brain network identified in Village L to predict attitudinal diversity scores of the Village K sample (n=47). As a result, connectivity of predictive mentalizing brain network as identified in Village L was positively correlated with ambivalence scores (r = 0.1424, p = 0.340) and standard deviation (r = 0.4042, p = 0.005) in Village K, revealing that our results may be successfully replicated in a completely independent sample.

**10. Moderating effects** To show that the results were consistent even when using continuous variables, we examined the moderating effects of Burt’s structural constraint (continuous) on the association between mentalizing brain connectivity (continuous) and attitudinal diversity of social network members (continuous). As shown in Table S7 and Fig. S11, occupation of a structural brokerage position marginally moderated the association between mentalizing brain functional connectivity and ambivalence scores (p=0.057). Also, a structural brokerage position significantly moderated the association between mentalizing brain functional connectivity and standard deviation (p=0.023). Consistent with the results in the Main Manuscript Social Correlates section, participants who were simultaneously in the high brain functional connectivity group (higher mentalizing brain connectivity) and the structural brokerage group (lower structural constraint) showed high ambivalence scores and standard deviations.

It should be noted that social network position was correlated with mentalizing brain connectivity in the previous literature (20). Therefore, In Table S7, we also tested two alternative model specifications. In the first alternative model, mentalizing was predicted by the interaction between attitudinal diversity and structural brokerage position (Burt’s structural constraint). The table confirms that the statistical fit measured by r-square was worse than the original model specification. Second, the model that predicted structural brokerage based on the interaction between mentalizing brain connectivity and attitudinal diversity was also tested. Similarly, the statistical fit was worse than the original model specification.

**11. Additional analyses for gender role attitudes** Considering that the reliability estimate of our gender role attitude measure was not so high (Spearman-Brown coefficient=0.222), we examined whether one of the two items in our measure drives our results. As described in the Materials and Methods section, gender role attitude measure consists of two items: “(1) Both the man and woman should contribute to the household income (first item)” and “(2) A man’s job is to earn money; a woman’s job is to look after the home and family (second item).” Using only one of the two items, we re-calculated attitudinal diversity scores of social network members. After that, in the regression model, we included “attitudinal diversity scores calculated using the first item” and “attitudinal diversity scores calculated using the second item” to predict mentalizing connectivity.

The results are shown in Table S9. As shown in the table, both kinds of ambivalence scores were significantly associated with mentalizing connectivity. The difference between the two coefficients was not statistically significant (p=0.668). Also, both kinds of standard deviation scores were significantly associated with mentalizing connectivity, and the difference between the two coefficients was not statistically significant (p=0.388). Therefore, it seemed that our study results were not driven by one particular item of gender role attitude measure.

Fig. S1. An illustration of a typical structural brokerage and structural closure position. Person A occupies an archetypal structural closure position while person B is on a structural brokerage position.


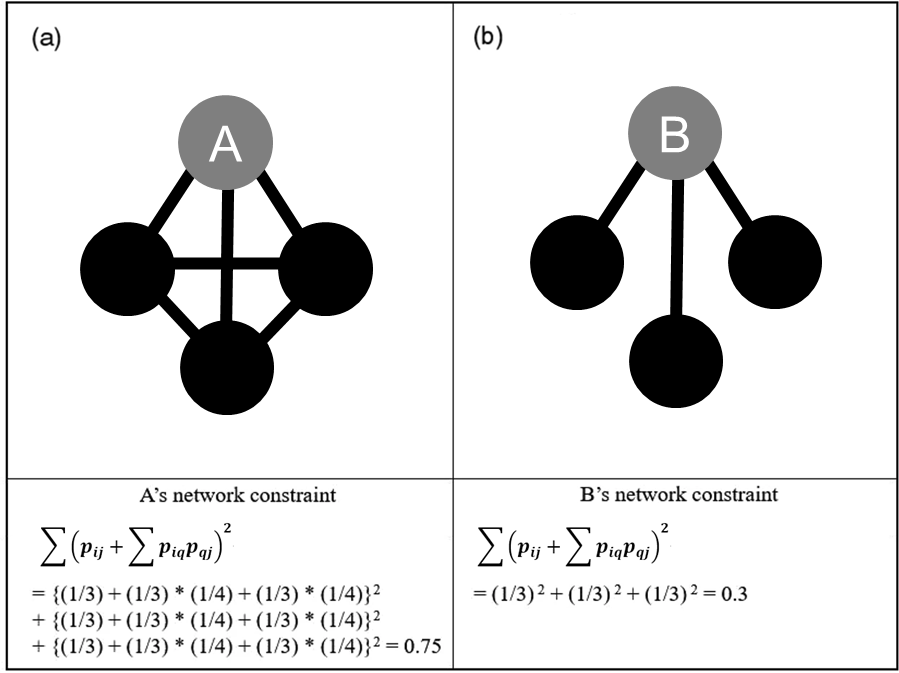


Fig. S2. Distribution of attitudinal diversity scores. Participants differed widely with respect to the attitudinal diversity of social network members.


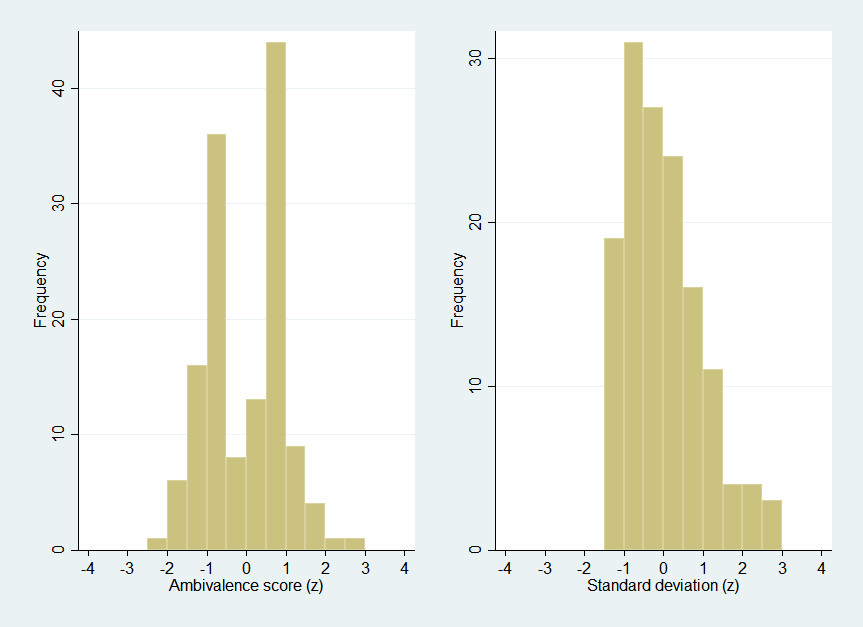


**Fig. S3.** The association between gender role attitude and attitudinal diversity scores (both ambivalence score and standard deviation). Traditional people look like they connect with more diverse others.

| (a) Ambivalence score |
| --- |
| 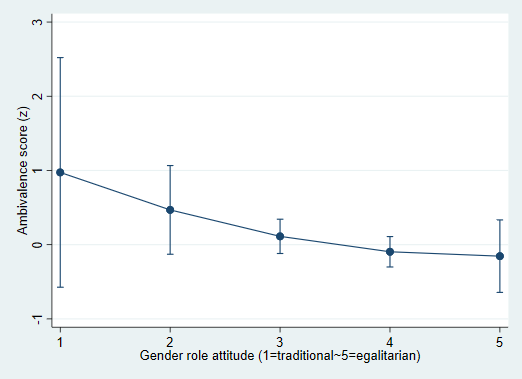 |
| (b) Standard deviation |
| 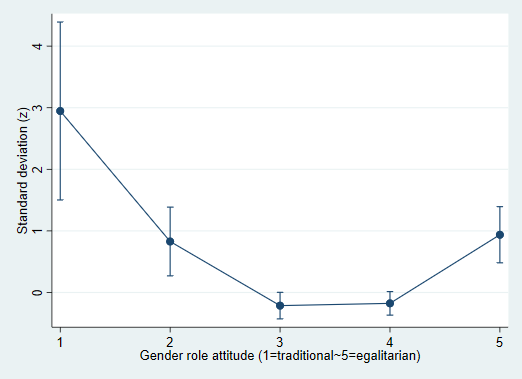 |

**Fig. S4.** Histogram of gender role attitude. There were more egalitarian people than traditional people in our sample


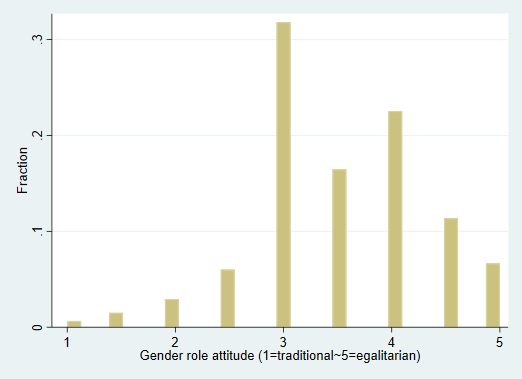


Fig. S5. Mentalizing regions (ROI) defined by Neurosynth meta-analytic tool. We identified 51 mentalizing-related brain regions among the 227 regions from Shen’s whole-brain parcellation atlas, based on the Neurosynth meta-analytic tool. L, left hemisphere; R, right hemisphere.


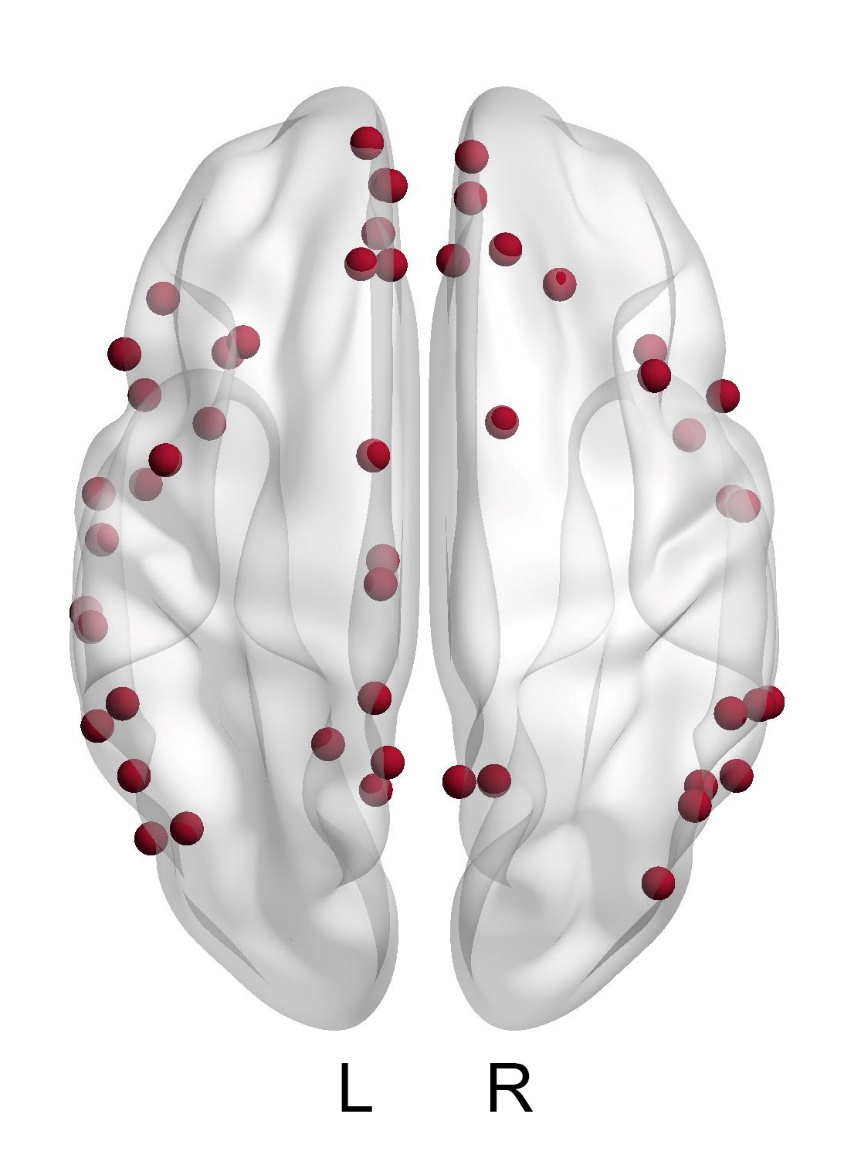


**Fig S6.** Leave-one-out cross-validation results. LOOCV revealed that mentalizing brain connectivity positively predicted ambivalence score (r = 0.2301, p = 0.046; MAE = 0.8305, p = 0.030) and standard deviation (r = 0.2033, p = 0.061; MAE = 0.8190, p = 0.114). On the other hand, neither ambivalence scores (r = 0.1136, p = 0.265; MAE = 0.8971, p = 0.274) nor standard deviations (r = -0.0182, p = 0.557; MAE = 0.9232, p = 0.712) were negatively predicted by mentalizing brain connectivity.

**
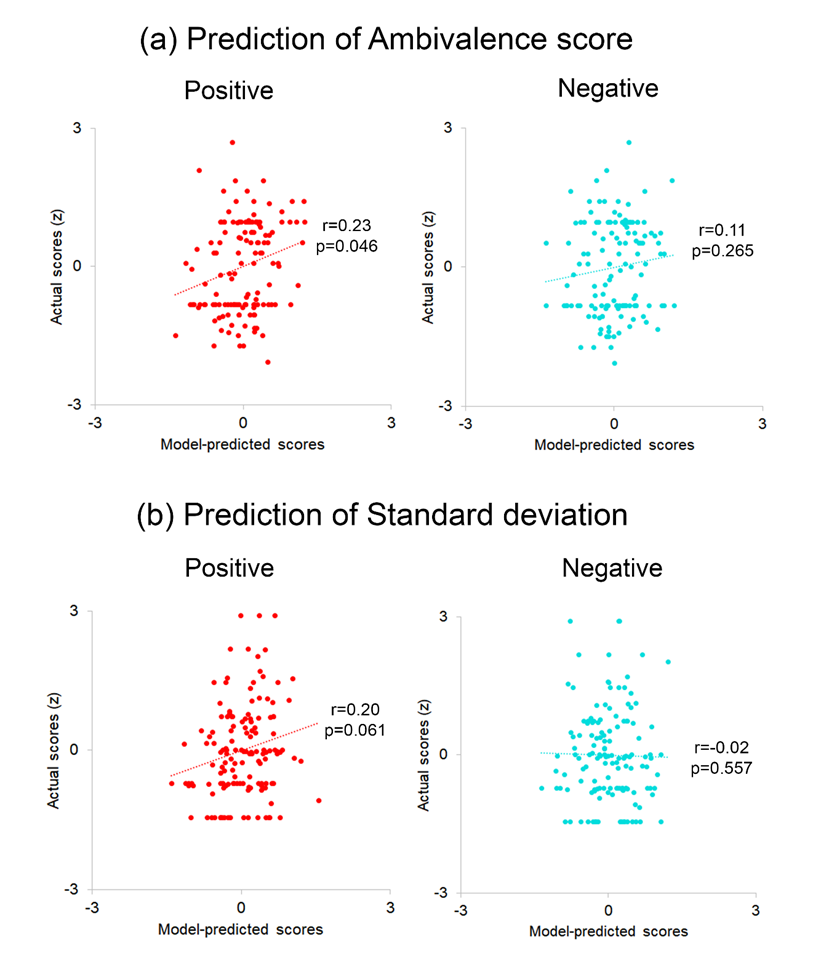
**

**Fig. S7.** Permutation distribution of prediction using 5-fold cross-validation. 5-fold cross-validation showed that both ambivalence scores (r = 0.2283, p = 0.011; MAE = 0.8368, p = 0.006) and standard deviations (r = 0.1912, p = 0.017; MAE = 0.8110, p = 0.018) were significantly and positively predicted by mentalizing brain connectivity. Each histogram depicts the distribution of predictive accuracy, *r*.

| (a) Prediction of Ambivalence score | |
| --- | --- |
| Positive | Negative |
| 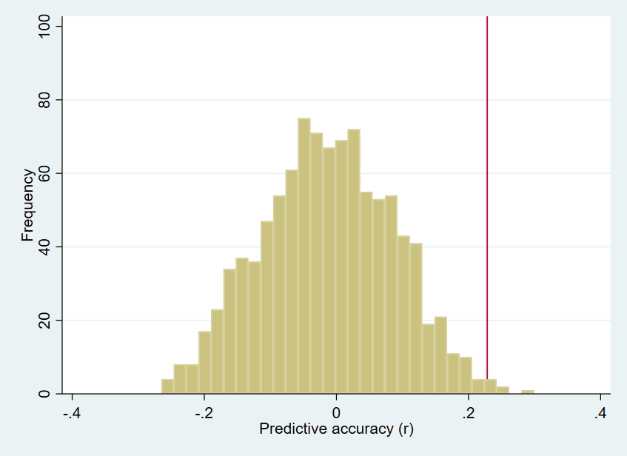 | 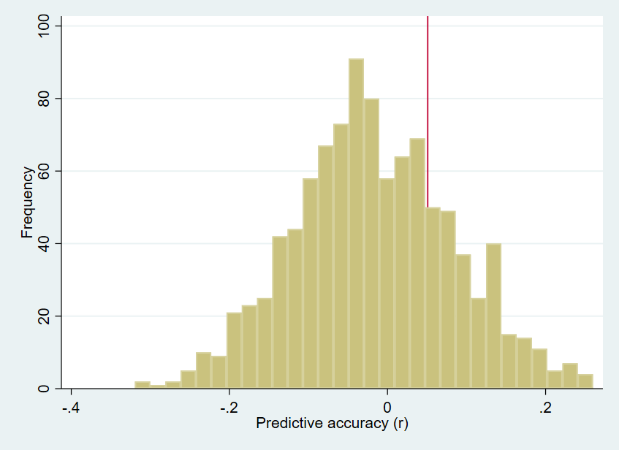 |
| (b) Prediction of Standard deviation | |
| Positive | Negative |
| 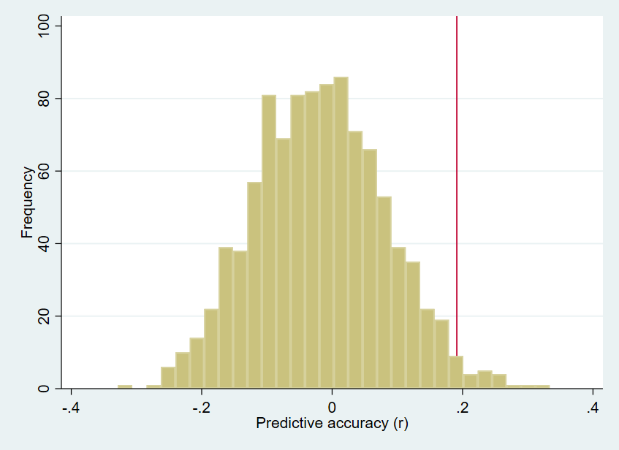 | 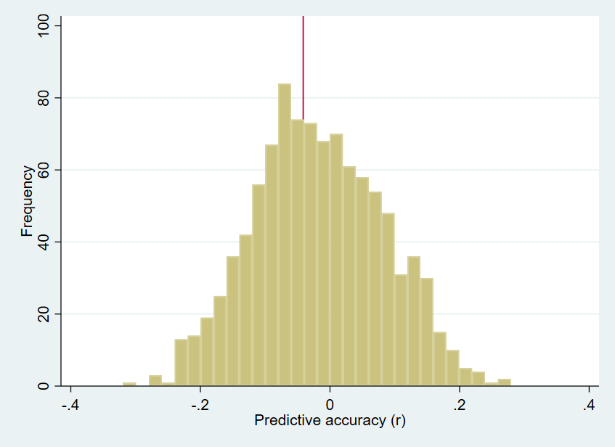 |

**Fig. S8.** Distribution of standardized score of the Burt’s structural constraint. Burt’s structural constraint was highly skewed.


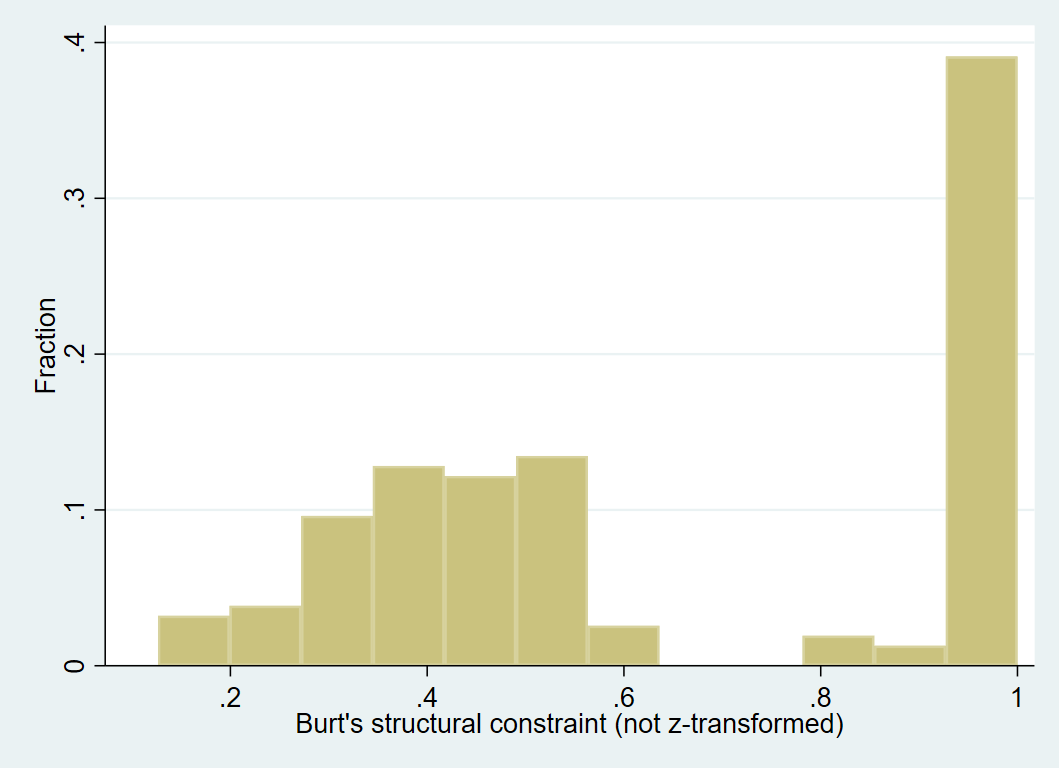


**Fig. S9.** Cluster of similar attitudes. These results indicate the strong homophily regarding the attitudes. We calculated how many egalitarian (traditional) people, on average, each egalitarian (traditional) person can reach out in the social network. The results showed that the observed numbers were much larger than those expected by chance.

Fig. S10. Depictions of the predictive mentalizing brain networks using Glass brains and circle plots. Colors within the circle plots correspond to lobes of the brain (Note that cerebellum lobe was not included). Red lines indicate the edges in the mentalizing brain connectivity that positively predicted ambivalence score or standard deviation. Yellow lines indicate the edges in the whole brain connectivity that positively predicted ambivalence score or standard deviation. L, left hemisphere; R, right hemisphere.


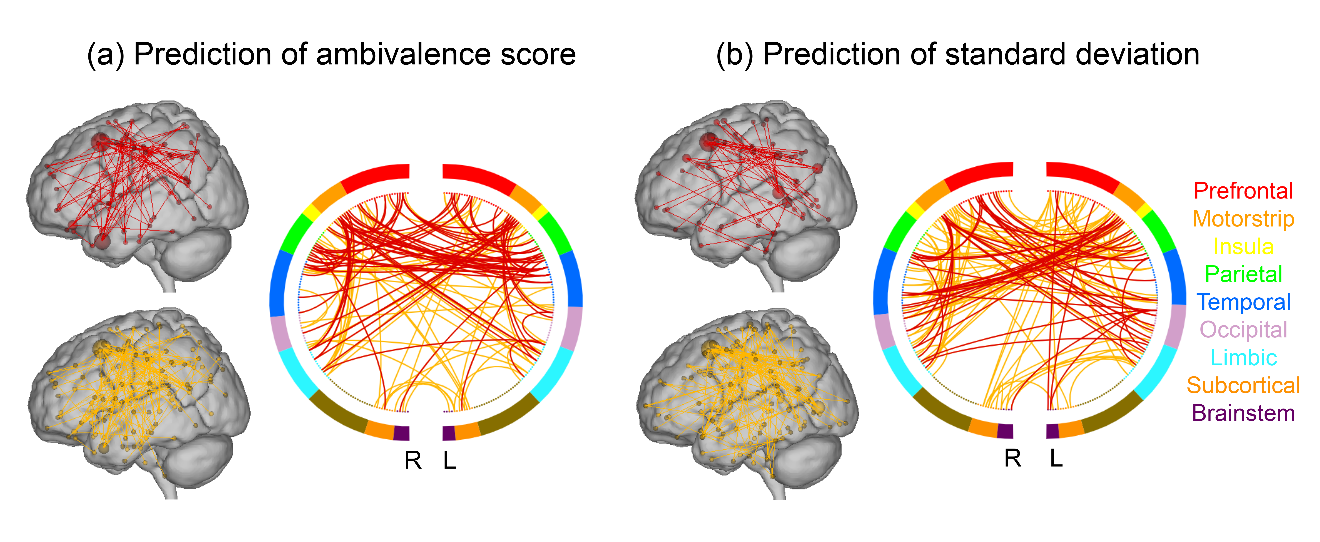


**Fig. S11.** Moderating analyses using continuous variables. Brokerage refers to structural brokerage (Burt’s structural constraint = mean - standard deviation) and Closure refers to structural closure (Burt’s structural constraint = mean + standard deviation)

| (a) Ambivalence score |
| --- |
| **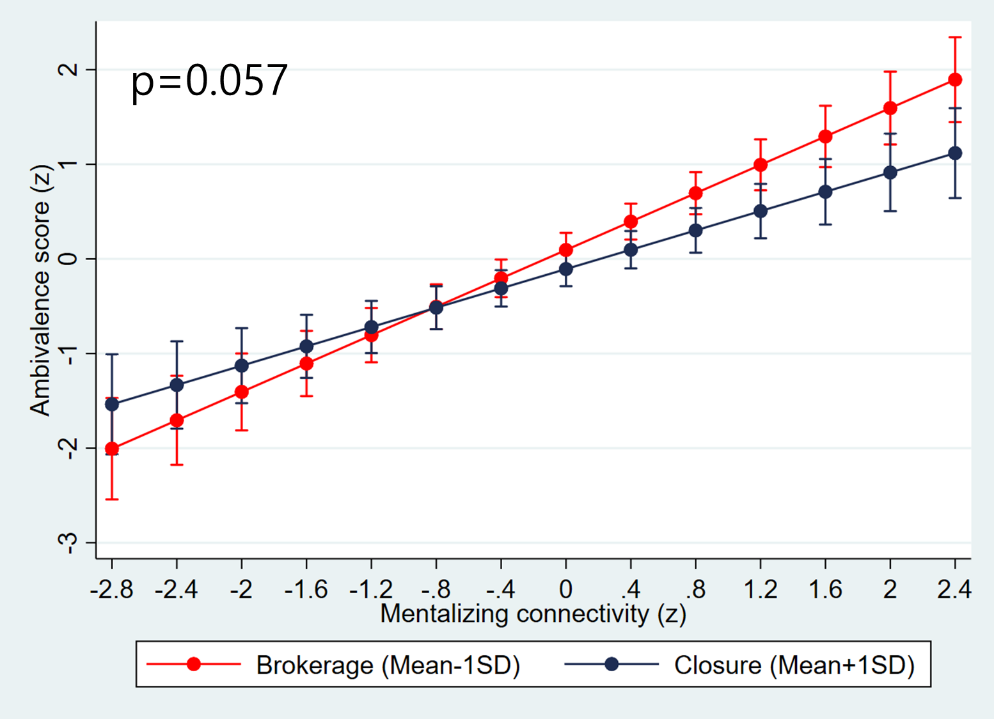** |
| (b) Standard deviation |
| **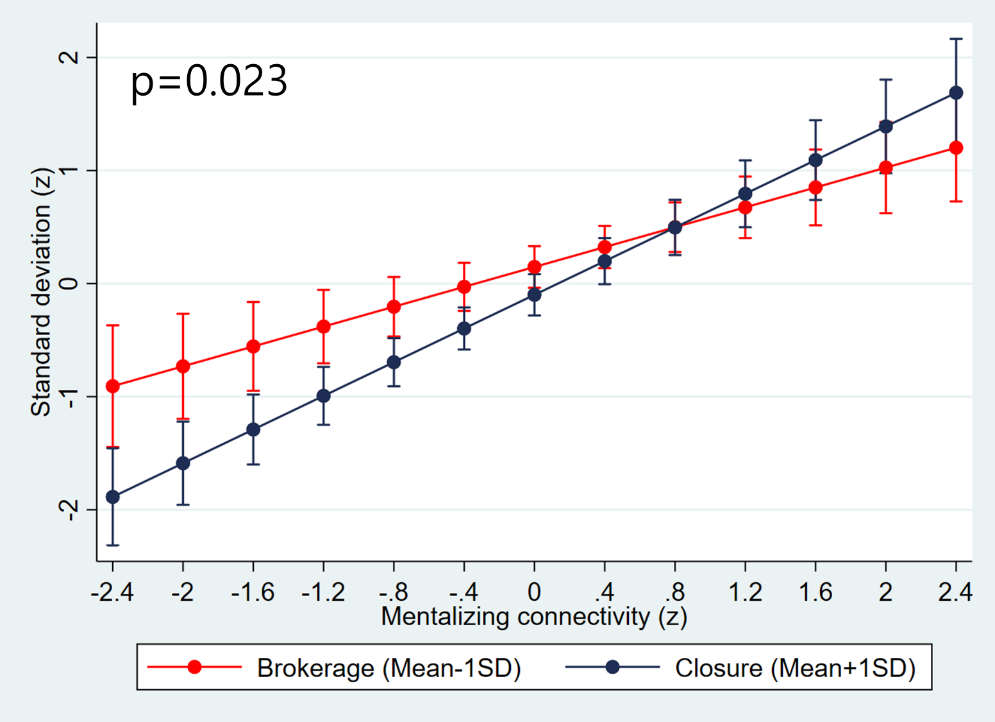** |

Table S1. Descriptive statistics

|  | Mean | SD | Min | Max |
| --- | --- | --- | --- | --- |
| Ambivalence score | -29.27 | 406.29 | -866.88 | 1069 |
| Standard deviation of gender role attitudes | 0.50 | 0.34 | 0 | 1.5 |
| Age (yrs) | 72.73 | 6.37 | 59 | 88 |
| Female | 0.55 | 0.50 | 0 | 1 |
| Education (yrs) | 7.55 | 4.33 | 0 | 24 |
| Gender role attitudes (1~5=egalitarian) | 3.67 | 0.76 | 1.50 | 5 |
| Village L | 0.66 | 0.47 | 0 | 1 |
| Social network characteristics |  |  |  |  |
| Social network size | 2.59 | 1.75 | 1 | 10 |
| Avg. communication freq. (days/yr) | 300.73 | 78.78 | 26 | 365 |
| Structural constraint | 0.66 | 0.30 | 0.13 | 1 |
| General health |  |  |  |  |
| MMSE (0~30) | 26.87 | 2.28 | 20 | 30 |
| Subjective health (1: Poor~5: Excellent) | 2.89 | 0.95 | 1 | 5 |
| Personality |  |  |  |  |
| Agreeableness | 36.82 | 4.86 | 24 | 45 |
| Extraversion | 28.42 | 6.69 | 12 | 42 |
| Neuroticism | 21.22 | 7.90 | 0 | 44 |
| Openness to experience | 29.11 | 7.15 | 10 | 46 |
| Conscientiousness | 37.57 | 4.61 | 26 | 48 |

**Table S2**. The association between gender role attitude and attitudinal diversity scores.

|  | Ambivalence score | | | | Standard deviation | | | | |
| --- | --- | --- | --- | --- | --- | --- | --- | --- | --- |
|  | Coef | p | Coef | p | Coef | p | Coef | p |
| Gender role attitudes | -0.199+ | 0.076 | -0.731 | 0.400 | 0.099 | 0.356 | -3.569 *** | 0.000 |
| Gender role attitudes^2^ |  |  | 0.075 | 0.537 |  |  | 0.515 *** | 0.000 |

+p<0.1, ***p<0.001

Table S3. Control analyses (positive prediction)

| Control variables | Prediction of ambivalence score | | | | Prediction of standard deviation | | | |
| --- | --- | --- | --- | --- | --- | --- | --- | --- |
|  | **r** | **p** | **MAE** | **p** | **r** | **p** | **MAE** | **p** |
| Age (yrs) + Female | 0.2294+ | 0.052 | 0.8328* | 0.036 | 0.2401* | 0.031 | 0.8017+ | 0.057 |
| Education (yrs) | 0.2372* | 0.048 | 0.8316* | 0.039 | 0.2465* | 0.030 | 0.8046+ | 0.070 |
| Social network characteristics |  |  |  |  |  |  |  |  |
| Social network size | 0.2475* | 0.037 | 0.8264* | 0.026 | 0.1835+ | 0.085 | 0.8195 | 0.112 |
| Avg. communication freq. (days/yr) | 0.2231+ | 0.055 | 0.8365* | 0.036 | 0.2196* | 0.046 | 0.8103+ | 0.092 |
| Structural constraint | 0.2824* | 0.019 | 0.8103* | 0.017 | 0.2120* | 0.048 | 0.8133+ | 0.088 |
| Betweenness centrality | 0.2374+ | 0.052 | 0.8300+ | 0.053 | 0.2273+ | 0.051 | 0.8114+ | 0.059 |
| Closeness centrality | 0.2635* | 0.027 | 0.8156* | 0.026 | 0.2582* | 0.013 | 0.8005* | 0.016 |
| Eigenvector centrality | 0.2463* | 0.038 | 0.8278* | 0.042 | 0.2637* | 0.018 | 0.7970* | 0.021 |
| MMSE (0~30) | 0.2377* | 0.045 | 0.8274* | 0.027 | 0.2089+ | 0.065 | 0.8186 | 0.115 |
| Subjective health (1=poor~5=excellent) | 0.2074+ | 0.075 | 0.8401* | 0.046 | 0.2370* | 0.032 | 0.8041+ | 0.068 |
| Personality |  |  |  |  |  |  |  |  |
| Agreeableness | 0.2251+ | 0.054 | 0.8343* | 0.034 | 0.2396* | 0.027 | 0.8047+ | 0.066 |
| Extraversion | 0.2864* | 0.017 | 0.8121* | 0.014 | 0.2561* | 0.018 | 0.7913* | 0.028 |
| Neuroticism | 0.2543* | 0.034 | 0.8276* | 0.028 | 0.2419* | 0.030 | 0.7963* | 0.037 |
| Openness to experience | 0.2498* | 0.039 | 0.8233* | 0.030 | 0.2188* | 0.043 | 0.8134+ | 0.094 |
| Conscientiousness | 0.2260+ | 0.056 | 0.8352* | 0.036 | 0.2772* | 0.010 | 0.7871* | 0.028 |
| Gender role attitudes (1=traditional~5=egalitarian) | 0.1867 | 0.103 | 0.8539+ | 0.084 | 0.2565* | 0.018 | 0.7977* | 0.045 |
| Head motion (max and mean FD) | 0.2439* | 0.044 | 0.8261+ | 0.053 | 0.1937+ | 0.065 | 0.8228 | 0.101 |
| Village L | 0.3009* | 0.013 | 0.8025** | 0.008 | 0.2198* | 0.040 | 0.8072+ | 0.064 |

**p<0.01 *p<0.05 +p<0.1

Age and sex were controlled by default.

Table S4. Neuroanatomy of mentalizing connectivity predicting ambivalence score (top 15 highest-degree nodes)

| Node | K | Node name | L/R | Lobe | Mentalizing region | Default mode network | MNI coordinates |
| --- | --- | --- | --- | --- | --- | --- | --- |
| 30 | 14 | Middle frontal gyrus | R | Motorstrip |  | yes | 25.22, 12.41, 49.39 |
| 187 | 12 | Anterior temporal lobe | L | Temporal | yes | yes | -49.49, 11.11, -30.56 |
| 3 | 7 | Ventromedial PFC | R | Prefrontal | yes | yes | 5.08, 34.90, -17.35 |
| 45 | 6 | Supramarginal gyrus (Inferior parietal lobe) | R | Parietal |  |  | 52.84, -27.25, 40.93 |
| 86 | 5 | Posterior cingulate cortex | R | Limbic | yes | yes | 12.34, -57.20, 18.11 |
| 164 | 5 | Superior frontal gyrus | L | Motorstrip |  | yes | -23.22, 10.66, 53.61 |
| 26 | 4 | Precentral gyrus | R | Motorstrip |  |  | 26.32, -12.91, 66.20 |
| 179 | 4 | Superior parietal lobule | L | Parietal |  |  | -35.72, -39.34, 47.75 |
| 182 | 4 | Temporoparietal junction | L | Parietal | yes | yes | -42.05, -65.62, 41.73 |
| 190 | 4 | Anterior temporal lobe | L | Temporal | yes | yes | -57.62, -6.37, -22.69 |
| 29 | 3 | Pre-SMA | R | Motorstrip | yes |  | 13.73, 6.27, 65.35 |
| 50 | 3 | Posterior superior temporal sulcus | R | Parietal | yes | yes | 48.95, -58.12, 14.36 |
| 53 | 3 | Temporal pole | R | Temporal | yes | yes | 52.84, 10.90, -21.83 |
| 90 | 3 | Precuneus cortex | R | Limbic | yes | yes | 6.17, -57.36, 38.15 |
| 145 | 3 | Dorsomedial PFC | L | Prefrontal | yes | yes | -10.15, 55.69, 30.24 |

Node, Node ID in Shen’s whole-brain atlas. K, degree; L, left; R, right; PFC, prefrontal cortex; SMA, supplementary motor area; Default mode network, whether each brain region belongs to the association test map of the term “default mode” from the Neurosynth meta-analytic tool.

Table S5. Neuroanatomy of mentalizing connectivity predicting standard deviation (top 15 highest-degree nodes)

| Node | K | Node name | L/R | Lobe | Mentalizing region | Default mode network | MNI coordinates |
| --- | --- | --- | --- | --- | --- | --- | --- |
| 30 | 9 | Middle frontal gyrus | R | Motorstrip |  | yes | 25.22, 12.41, 49.39 |
| 65 | 6 | Posterior superior temporal sulcus | R | Temporal | yes |  | 59.25, -43.77, 8.46 |
| 49 | 5 | Intraparietal sulcus | R | Parietal | yes | yes | 41.39, -75.34, 27.98 |
| 205 | 5 | Posterior cingulate cortex (Lingual gyrus) | L | Occipital | yes | yes | -17.04, -50.71, 0.78 |
| 13 | 4 | Dorsolateral PFC | R | Prefrontal | yes | yes | 23.92, 30.67, 36.41 |
| 164 | 4 | Superior frontal gyrus | L | Motorstrip |  | yes | -23.22, 10.66, 53.61 |
| 184 | 4 | Supramarginal gyrus (Inferior parietal lobe) | L | Parietal | yes | yes | -53.42, -43.53, 38.77 |
| 14 | 3 | Dorsolateral PFC | R | Prefrontal | yes | yes | 40.68, 14.51, 48.21 |
| 29 | 3 | Pre-SMA | R | Motorstrip | yes |  | 13.73, 6.27, 65.35 |
| 162 | 3 | Pre-SMA | L | Motorstrip | yes |  | -9.06, 0.38, 66.53 |
| 201 | 3 | Parietal operculum cortex | L | Temporal |  |  | -46.67, -39.97, -24.30 |
| 48 | 2 | Temporoparietal junction | R | Parietal | yes | yes | 47.83, -61.58, 34.71 |
| 79 | 2 | Lingual gyrus | R | Occipital |  |  | 6.99, -75.69, -2.84 |
| 86 | 2 | Precuneus cortex | R | Limbic | yes | yes | 12.34, -57.20, 18.11 |
| 165 | 2 | Precentral gyrus | L | Motorstrip | yes |  | -45.80, -0.43, 49.28 |

Node, Node ID in Shen’s whole-brain atlas. K, degree; L, left; R, right; PFC, prefrontal cortex; SMA, supplementary motor area; MedF, Medial frontal; FP, Frontoparietal; DMN, Default mode; Sub, Subcortical-cerebellum; Mo, Motor; Mvis, Visual-medial; Lvis, Visual-lateral; VA, Visual association; Default mode network, whether each brain region belongs to the association test map of the term “default mode” from the Neurosynth meta-analytic tool.

**Table S6**. Correlation coefficients.

|  | (1) | (2) | (3) | (4) | (5) | (6) | (7) | (8) | (9) |
| --- | --- | --- | --- | --- | --- | --- | --- | --- | --- |
| (1) Ambivalence score |  |  |  |  |  |  |  |  |  |
|  |  |  |  |  |  |  |  |  |  |
| (2) Standard deviation of gender role attitudes | 0.610* |  |  |  |  |  |  |  |  |
|  | (0.000) |  |  |  |  |  |  |  |  |
| (3) Mentalizing connectivity | 0.639* | 0.414* |  |  |  |  |  |  |  |
|  | (0.000) | (0.000) |  |  |  |  |  |  |  |
| (4) Gender role attitudes | -0.151 | 0.079 | -0.113 |  |  |  |  |  |  |
|  | (0.076) | (0.357) | (0.185) |  |  |  |  |  |  |
| (5) Network size | 0.220* | 0.220* | 0.096 | -0.071 |  |  |  |  |  |
|  | (0.009) | (0.009) | (0.263) | (0.405) |  |  |  |  |  |
| (6) Avg. communication freq. (days/yr) | 0.022 | 0.050 | 0.013 | 0.075 | -0.295* |  |  |  |  |
|  | (0.795) | (0.557) | (0.879) | (0.381) | (0.000) |  |  |  |  |
| (7) Structural constraint | -0.139 | -0.215* | -0.055 | 0.077 | -0.848* | 0.326* |  |  |  |
|  | (0.104) | (0.011) | (0.524) | (0.366) | (0.000) | (0.000) |  |  |  |
| (8) Betweenness centrality | 0.084 | 0.083 | 0.033 | 0.019 | 0.553* | -0.257* | -0.420* |  |  |
|  | (0.328) | (0.333) | (0.703) | (0.827) | (0.000) | (0.002) | (0.000) |  |  |
| (9) Closeness centrality | 0.067 | 0.110 | 0.037 | 0.000 | 0.534* | -0.195* | -0.636* | 0.380* |  |
|  | (0.432) | (0.197) | (0.665) | (0.997) | (0.000) | (0.021) | (0.000) | (0.000) |  |
| (10) Eigenvector centrality | 0.043 | 0.122 | 0.136 | -0.026 | 0.230* | 0.067 | -0.188* | 0.019 | 0.121 |
|  | (0.613) | (0.151) | (0.112) | (0.765) | (0.006) | (0.437) | (0.027) | (0.829) | (0.156) |

*p<0.05. P-values are reported in parenthesis.

**Table S7**. Moderating analyses

|  | Ambivalence score (z) | | Mentalizing connectivity (z) | | Burt's structural constraint (z) | |
| --- | --- | --- | --- | --- | --- | --- |
|  | Coef | p | Coef | p | Coef | p |
| Mentalizing connectivity (z) | 0.630*** | 0.000 |  |  | 0.044 | 0.690 |
| Burt's structural constraint (z) | -0.101 | 0.123 | 0.033 | 0.617 |  |  |
| Ambivalence score (z) |  |  | 0.654*** | 0.000 | -0.153 | 0.165 |
| Mentalizing connectivity X Burt's structural constraint | -0.120+ | 0.057 |  |  |  |  |
| Mentalizing connectivity X Ambivalence score |  |  |  |  | -0.179+ | 0.066 |
| Structural constraint X Ambivalence score |  |  | 0.037 | 0.573 |  |  |
| R-square | 0.434 | | 0.411 | | 0.045 | |
|  | Standard deviation (z) | | Mentalizing connectivity (z) | | Burt's structural constraint (z) | |
|  | Coef | p | Coef | p | Coef | p |
| Mentalizing connectivity (z) | 0.592*** | 0.000 |  |  | 0.037 | 0.725 |
| Burt's structural constraint (z) | -0.123+ | 0.063 | -0.018 | 0.792 |  |  |
| Standard deviation (z) |  |  | 0.648*** | 0.000 | -0.363** | 0.002 |
| Mentalizing connectivity X Burt's structural constraint | 0.153* | 0.023 |  |  |  |  |
| Mentalizing connectivity X Standard deviation |  |  |  |  | 0.302*** | 0.000 |
| Structural constraint X Standard deviation |  |  | -0.050 | 0.479 |  |  |
| R-square | 0.433 | | 0.401 | | 0.138 | |

***p<0.001 **p<0.01 *p<0.05 +p<0.1

**Table S8**. Mentalizing and lesioned connectivity analyses

|  | Ambivalence score (z) | | Standard deviation (z) | |
| --- | --- | --- | --- | --- |
|  | Coef | p | Coef | p |
| Mentalizing connectivity (z) | 0.266* | 0.014 | 0.133 | 0.212 |
| Lesioned connectivity (z) | -0.058 | 0.593 | 0.116 | 0.275 |
|  | | | | |
|  | F score | p | F score | p |
| Difference between the coefficients (two-tailed) | 2.79+ | 0.097 | 0.01 | 0.929 |

*p<0.05 +p<0.1

**Table S9**. Prediction of mentalizing connectivity based on attitudinal diversity scores (ambivalence score and standard deviation) calculated using only one item of our gender role attitude measure

|  | Mentalizing connectivity (z) | |
| --- | --- | --- |
|  | Coef | p |
| **Model 1.** |  |  |
| Ambivalence score using the first item (z) | 0.270** | 0.001 |
| Ambivalence score using the second item (z) | 0.224** | 0.007 |
|  | F score | p |
| Difference between the coefficients (two-tailed) | 0.18 | 0.668 |
|  |  |  |
| **Model 2.** |  |  |
| Standard deviation using the first item (z) | 0.315*** | 0.000 |
| Standard deviation using the second item (z) | 0.415*** | 0.000 |
|  | F score | p |
| Difference between the coefficients (two-tailed) | 0.75 | 0.388 |

***p<0.001 **p<0.01 *p<0.05 +p<0.1

**References**

1. Behzadi Y, Restom K, Liau J, Liu TT. A component based noise correction method (CompCor) for BOLD and perfusion based fMRI. Neuroimage. 2007;37(1):90-101.

2. Muschelli J, Nebel MB, Caffo BS, Barber AD, Pekar JJ, Mostofsky SH. Reduction of motion-related artifacts in resting state fMRI using aCompCor. Neuroimage. 2014;96:22-35.

3. Ciric L, Hussain N, Cakic N. Common fixed points for Ciric type f-weak contraction with applications. Publ Math(Debr). 2010;76(1-2):31-49.

4. Murphy K, Birn RM, Handwerker DA, Jones TB, Bandettini PA. The impact of global signal regression on resting state correlations: are anti-correlated networks introduced? Neuroimage. 2009;44(3):893-905.

5. Chai XJ, Castañón AN, Öngür D, Whitfield-Gabrieli S. Anticorrelations in resting state networks without global signal regression. Neuroimage. 2012;59(2):1420-8.

6. Shen X, Tokoglu F, Papademetris X, Constable RT. Groupwise whole-brain parcellation from resting-state fMRI data for network node identification. Neuroimage. 2013;82:403-15.

7. Yarkoni T, Poldrack RA, Nichols TE, Van Essen DC, Wager TD. Large-scale automated synthesis of human functional neuroimaging data. Nature methods. 2011;8(8):665.

8. Wasylyshyn N, Hemenway Falk B, Garcia JO, Cascio CN, O’donnell MB, Bingham CR, et al. Global brain dynamics during social exclusion predict subsequent behavioral conformity. Social cognitive and affective neuroscience. 2018;13(2):182-91.

9. Shen X, Finn ES, Scheinost D, Rosenberg MD, Chun MM, Papademetris X, et al. Using connectome-based predictive modeling to predict individual behavior from brain connectivity. Nature Protocols. 2017;12(3):506.

10. Feng C, Yuan J, Geng H, Gu R, Zhou H, Wu X, et al. Individualized prediction of trait narcissism from whole‐brain resting‐state functional connectivity. Human brain mapping. 2018.

11. Hsu W-T, Rosenberg MD, Scheinost D, Constable RT, Chun MM. Resting-state functional connectivity predicts neuroticism and extraversion in novel individuals. Social cognitive and affective neuroscience. 2018;13(2):224-32.

12. Rosenberg MD, Hsu W-T, Scheinost D, Todd Constable R, Chun MM. Connectome-based models predict separable components of attention in novel individuals. Journal of cognitive neuroscience. 2018;30(2):160-73.

13. Beaty RE, Kenett YN, Christensen AP, Rosenberg MD, Benedek M, Chen Q, et al. Robust prediction of individual creative ability from brain functional connectivity. Proceedings of the National Academy of Sciences. 2018:201713532.

14. Lin Q, Rosenberg MD, Yoo K, Hsu TW, O'Connell TP, Chun MM. Resting-State Functional Connectivity Predicts Cognitive Impairment Related to Alzheimer's Disease. Frontiers in aging neuroscience. 2018;10:94.

15. Legates DR, McCabe Jr GJ. Evaluating the use of “goodness‐of‐fit” measures in hydrologic and hydroclimatic model validation. Water resources research. 1999;35(1):233-41.

16. Madhyastha TM, Koh N, Day TK, Hernández-Fernández M, Kelley A, Peterson DJ, et al. Running Neuroimaging Applications on Amazon Web Services: How, When, and at What Cost? Frontiers in neuroinformatics. 2017;11:63.

17. Tang H, Lu X, Cui Z, Feng C, Lin Q, Cui X, et al. Resting-state functional connectivity and deception: exploring individualized deceptive propensity by machine learning. Neuroscience. 2018;395:101-12.

18. Han JW, Kim TH, Jhoo JH, Park JH, Kim JL, Ryu SH, et al. A normative study of the Mini-Mental State Examination for Dementia Screening (MMSE-DS) and its short form (SMMSE-DS) in the Korean elderly. Journal of Korean Geriatric Psychiatry. 2010;14(1):27-37.

19. Costa Jr T. The NEO-PI-R professional manual: Revised NEO Five-Factor Inventory.(NEO-FFI). Psychological assessment resources. 1992.

20. O’donnell MB, Bayer JB, Cascio CN, Falk EB. Neural bases of recommendations differ according to social network structure. Social cognitive and affective neuroscience. 2017;12(1):61-9.

21. Feng C, Yuan J, Geng H, Gu R, Zhou H, Wu X, et al. Individualized prediction of trait narcissism from whole‐brain resting‐state functional connectivity. Human brain mapping. 2018;39(9):3701-12.
